# Radical Scavenger Capacity of Jabuticaba Fruit (*Myrciaria cauliflora*) and Its Biological Effects in Hypertensive Rats

**DOI:** 10.1155/2017/2383157

**Published:** 2017-12-20

**Authors:** Camila Gabriela de Souza, Daniela Medeiros Lobo de Andrade, Juliana Bahia Reis Jordão, Renato Ivan de Ávila, Leonardo Luiz Borges, Boniek Gontijo Vaz, Marize Campos Valadares, Eric de Souza Gil, Edemilson Cardoso da Conceição, Matheus Lavorenti Rocha

**Affiliations:** ^1^Faculty of Pharmacy, Federal University of Goiás, Avenida Universitária s/n, 74605-220 Goiânia, GO, Brazil; ^2^Unit of Exact and Technological Sciences, State University of Goiás, Caixa Postal 459, 75132-903 Anápolis, GO, Brazil; ^3^School of Medical, Pharmaceutical and Biomedical Sciences, Pontifical Catholic University of Goiás, 74605-010 Goiânia, GO, Brazil; ^4^Institute of Chemistry, Federal University of Goiás, Campus Samambaia, Caixa Postal 131, 74001-970 Goiânia, GO, Brazil

## Abstract

Jabuticaba is an exotic fruit native to Brazil that has been arousing medicinal interest. Using chemical (HPLC-PDA, resonance mass spectra, and NMR), electroanalytical (differential pulse voltammetry, radical scavenging assay), and pharmacological (*in vivo* and *in vitro*) approaches, we have identified its bioactive compounds and hypotensive effects on hypertensive rats. The hydroalcoholic extract of jabuticaba (HEJ) presents a great quantity of phenolic compounds, and several molecules with hydroxyl groups present high efficiency as an antioxidant. The treatment with HEJ (100 and 300 mg/kg/day, for four weeks) presented hypotensive effects on L-NAME-induced hypertensive rats, possibly improving the nitric oxide bioavailability because of its high antioxidant potential. Furthermore, renal and cardiac hypertrophies were also attenuated after the HEJ treatment. Moreover, the vascular responses to contractile and dilating agonists were improved with the HEJ treatment, which is also able to induce nitric oxide production in endothelial cells.

## 1. Introduction

Hypertension is the main risk factor for the development of cardiovascular diseases, accounting for approximately 10 million deaths per year [1]. To control vascular tone and consequently regulate blood pressure, the endothelium produces several substances with vasodilatory action. The main one is nitric oxide (NO), produced through the reaction catalyzed by the enzyme NO synthase (NOS). NO controls various physiological and pathological processes in the cardiovascular system, in addition to contributing directly to the regulation of blood pressure [2]. Any inhibition of NO synthesis or bioactivity impairs vascular relaxation and blood pressure control, leading to systemic hypertension. In experimental models, NO levels can be suppressed using NOS inhibitors (such as L-NAME), which cause systemic hypertension comparable to hypertension observed in humans, causing several cardiovascular changes and increasing reactive oxygen species (ROS) [3].

The use of natural substances has been gaining more attention in the treatment of cardiovascular diseases, and several plant species have shown important hypotensive results with few adverse effects [4, 5]. Jabuticaba is a native Brazilian fruit (Myrtaceae) that has the same shape and size as grapes and a color that ranges from a deep purple to black. Like grapes, jabuticaba also perishes quickly; hence, it must be consumed shortly after harvest. This fruit is very popular in Brazil and is often used in the production of jams, ice creams, juices, and alcoholic beverages, which have been increasing in consumption in Brazil and abroad [6, 7]. It has a pleasant flavor, and its taste is sweet with a slightly sour accent. In addition to its use as a food and beverage, jabuticaba is used in folk medicine (mainly in south Brazil) for its antioxidant benefits and for treating spasmodic vasomotor disturbances [8, 9].

Several phenolic compounds, flavonoids, and anthocyanins are present in the peel of the fruit, which may be responsible for a number of biological effects of this species and has been of great medicinal interest [10, 11]. These compounds are capable of complexing free radicals and inhibiting the initiation of the chain of propagation of oxidative reactions, including lipid peroxidation [12].

Our research group has shown important effects of this species on the cardiovascular system *in vitro*, such as the vascular dilatatory effect acting on the endothelial cells of the vessels [13] or a direct relaxing effect on vascular smooth muscle, activating K^+^ channels and inhibiting Ca^2+^ influx [14]. In this work, we chemically studied the electroanalytical profile of this species and the antioxidant capacities and effects of chronic treatment with jabuticaba berry in hypertensive animals to associate the cardiovascular effects with the typical phytochemistry groups present in jabuticaba.

## 2. Materials and Methods

### 2.1. Plant Material and Preparation of Extract

Mature jabuticaba fruits (*Myrciaria cauliflora* (Mart.) O. Berg) were donated by “Jaboticabal” winery in the city of Hidrolândia, GO, Brazil (16°57′57^″^S and 49°13′35^″^W). A voucher specimen (number 21140) has been deposited in the herbarium of the ICB/UFG Botany Department. The seedless fruits were air-dried (40°C), pulverized in a knife mill, and passed through a 60-mesh sieve in the Laboratory of Natural Products Research, Faculty of Pharmacy, Federal University of Goiás. The obtained powder product underwent exhaustive percolation with ethanol : water (55 : 45, *v*/*v*). The extract obtained was filtered and evaporated under reduced pressure in a rotary evaporator at 40°C to provide the hydroalcoholic extract of jabuticaba (HEJ) with a yield of ~8.56%. The HEJ was dissolved in distilled water at a concentration of 120 mg/mL and stored at −20°C without contact with the clarity until the experiments.

### 2.2. Phytochemical Analyses

For HPLC-PDA analyses of the HEJ, ellagic acid was used as the internal phytochemical standard [6, 15]. These analyses were carried out using the Waters LC system (Milford, Massachusetts, USA) comprising a quaternary pump, an online degasser, an autosampler, and a photodiode array detector model 2998. Empower 2.0 software was used for the control of the HPLC equipment and for the acquisition and treatment of data. Chromatographic separation was carried out with a C18 reverse phase column (250 × 4.6 mm, 5 *μ*m) purchased from Phenomenex (Phenomenex Inc., Torrance, CA, USA). The detection wavelength was 252 nm at a flow rate of 0.5 mL/min at 25°C in 10 min (mobile phase was composed of methanol : water (60 : 40, *v*/*v*)).

A colorimetric test was carried out to verify the total phenolic compounds in HEJ, using ferric chloride in aqueous extract solution under alkaline conditions to result in a colored complex with phenolic compounds, read at 510 nm [16].

In order to elucidate the compounds present in the extract, the HEJ was analyzed by ESI FT-ICR mass spectra. Briefly, the HEJ sample was diluted to ≈0.25 mg/mL in water/methanol (1 : 1, *v*/*v*) which contained 0.1% (*m*/*v*) of NH_4_OH for ESI in a negative mode. The resulting solution was directly infused at a flow rate of 3 *μ*L/min into the ESI source. The mass spectrometer (model 9.4T Solarix, Bruker Daltonics, Bremen, Germany) was set to operate over a mass range of *m*/*z* 150–2000. The ESI source conditions were as follows: a nebulizer gas pressure of 3 bar, a capillary voltage of 3.5 kV, and a transfer capillary temperature of 250°C. The ions are accumulated in the hexapolar collision cell with a time of 5.10^−3^ s followed by transport to the analyzer cell (ICR) through the multipole ion guide system (another hexapole). The time of flight in the hexapole was 0.5 ms. Each spectrum was acquired by accumulating 200 scans of time-domain transient signals in four megapoint time-domain data sets. All mass spectra were externally calibrated using sodium trifluoroacetate solutions (*m*/*z* from 200 to 2000). A resolving power, *m*/Δm50% ≅ 730,000, in which Δm50% is the full peak width at the half-maximum peak height, of *m*/*z* 6400 and a mass accuracy of <1 ppm provided the unambiguous molecular formula assignments for singly charged molecular ions. The mass spectra were acquired and processed using data analysis software (Bruker Daltonics, Bremen, Germany). The MS data were processed, and the elemental compositions of the compounds were determined by measuring the *m*/*z* values. The proposed structures for each formula were assigned using the ChemSpider (http://www.chemspider.com) database.

Nuclear magnetic resonance (NMR) analyses were also performed. All ^1^H NMR analyses were performed on a Bruker Avance III 500 11.75 Tesla spectrometer, at 298 K, using a 5 mm inverse probe head. The spectra were obtained at 500.13 MHz for ^1^H, using lyophilized HEJ and a solution of 600 *μ*L of D_2_O and 0.1% sodium-3-trimethylsilylpropionate (TMSP-2,2,3,3-d4) (*m*/*v*). D_2_O/TMSP was used as field frequency lock and internal standard. Sixty-four pulses were employed on the acquisition of the spectra, with an acquisition time of 3.28 s, a spectral width of 10,000 Hz, and a relaxation delay of 1 s. The NOESYGPPR1D sequence was applied for water signal suppression, with a mixing time of 150 ms. For quantitative analysis, 0.3 *μ*L of dimethylformamide, 600 *μ*L of D_2_O/TMSP solution, and 18.6 mg of sample were used. The experiments were done in triplicate. The signal area of DMF was measured and compared with those of the compounds in the extract to determine the absolute concentrations of those compounds. The relaxation delay was set to five times the value of the longest spin–lattice relaxation time T1 of the integrated resonance signals in order to ensure full relaxation of the corresponding protons.

### 2.3. Antioxidant Activity

#### 2.3.1. Electroanalytical Assay (Differential Pulse Voltammetry (DPV))

These experiments were carried out according to previously standardized methods [17]. Voltammetric experiments were performed in a potentiostat/galvanostat *μ*Autolab III® integrated to the GPES 4.9® software (Eco Chemie, Utrecht, The Netherlands). Measurements were performed using 50 *μ*L of HEMC (120 mg/mL) in 0.1 M phosphate buffer solution (pH 6.0) in a 5.0 mL one-compartment electrochemical cell, with a three-electrode system consisting of a carbon paste electrode (prepared as a piston-driven holder containing 70% of graphite powder and 30% of purified mineral oil, *Ø* = 2 mm), a Pt wire, and the Ag/AgCl/KCl_sat_ (both purchased from Analyser, São Paulo, Brazil), representing the working electrode, the counter electrode, and the reference electrode, respectively. The surface of the carbon paste electrode was mechanically renewed before the start of a new set of experiments by extruding approximately 0.5 mm of carbon paste out of the electrode holder and smoothing it with a filter paper. This procedure ensured very reproducible experimental results.

The experimental conditions for differential pulse voltammetry (DPV) were as follows: pulse amplitude, 50 mV; pulse width, 0.4 s; and scan rate, 5 mV·s^−1^. All experiments were done at room temperature (21 ± 1°C) in triplicate (*n* = 3) and treated with the software Origin 8^®^.

#### 2.3.2. DPPH Radical Scavenging Assay

The antioxidant capacity of different extracts was evaluated by means of the conversion (decolorization) of stable 1,1-diphenyl-2-picrylhydrazyl (DPPH·) radical into its reduced form (DPPH) in accordance with the procedure revised and described by Lino et al. [17]. Briefly, a reaction solution consisting of 2.5 mL of 0.1 mM DPPH ethanolic solution was mixed to an aliquot of 0.5 mL of ethanol in order to reach *A*~0.7 at *λ* = 517 nm, whereas the ethanol, the solvent used to prepare all solutions, was used in order to adjust the baseline (*A* = 0.000). In turn, the same amount of sample and standard ethanolic solutions were added to other reaction systems, and the antioxidant activity was expressed as gallic acid equivalents.

### 2.4. Animals

Male Wistar rats (200–230 g) from the Central Animal House, at the Federal University of Goiás, were used for the experiments. The animals were housed in a temperature- and light-controlled room (22 ± 2°C; 12 h light/dark cycle) with free access to water and rodent chow and acclimatized for a period of at least one week before starting the experiment. They were handled in accordance with the internationally accepted standard guidelines for the use of animals. All procedures were approved by the Animal Research Ethics Committee at the Federal University of Goiás, Goiânia, Brazil (protocol: 015/2014).

The hypertension was induced by oral administration with L-NAME (60 mg/kg/day in drinking water) for six weeks. HEJ was administered orally (100 or 300 mg/kg/day by gavage in distilled water) starting at the second week along with oral treatment with L-NAME until the end of the sixth week. The control group received only drinking water and vehicle (distilled water). The rats were weighed weekly, and the systolic blood pressure (SBP) and heart rate (beats per min (bpm)) were also measured once a week using noninvasive tail-cuff plethysmography (Panlab Harvard Apparatus, Barcelona, Spain).

### 2.5. Studies in Isolated Arteries

Following the hemodynamic measurements in the sixth week, rats were anaesthetized (inhaled isoflurane) and killed by cervical dislocation. The heart and left kidney were isolated and weighed in relation to the corporal weight. The thoracic aorta was isolated, cleaned, and cut into rings approximately 4 mm in length; placed in an organ bath between two stainless-steel stirrups; and connected to a computerized system and a WinDaq Resource (DATAQ Instruments, Akron, OH, USA) data acquisition unit to measure isometric tension in the preparations. The aortic rings were placed in a 10 mL organ chamber containing a Krebs solution of the following compositions: 130 mM NaCl, 4.7 mM KCl, 1.2 mM KH_2_PO_4_, 1.2 mM MgSO_4_, 14.9 mM NaHCO_3_, 5.5 mM glucose, and 1.6 mM CaCl_2_ (pH 7.4), at 36°C and gassed with 95% O_2_ and 5% CO_2_. The rings were initially stretched to a basal tension of 1.5 g before allowing them to equilibrate in the bathing medium. Each rat supplied only one aortic ring for different protocols.

In some preparations, the endothelial cells were mechanically removed by rubbing the internal artery surface with a fine metallic wire and the effectiveness of the removal was demonstrated by the absence of relaxation to acetylcholine (ACh) (1 *μ*M) after being precontracted with phenylephrine (Phe) (0.1 *μ*M). For studies in preparations with the endothelium, the rings were discarded when the relaxation to ACh was less than 80%. Each ring was sequentially washed, reequilibrated, and then left to relax. Again, the aortic rings with or without the endothelium were contracted with Phe (0.1 *μ*M), and cumulative concentration-response curves for ACh (0.1 nM to 10 *μ*M) or sodium nitroprusside (SNP) (0.01 nM to 1 *μ*M) were carried out. In another series of experiments, cumulative concentration-response curves were carried out for phenylephrine (Phe) (0.1 nM a 10 *μ*M) in aortic rings without the endothelium.

### 2.6. Evaluation of Cytosolic Nitric Oxide ([NO]_c_) Levels in Endothelial Cells

After isolation, the arteries were sectioned longitudinally and the endothelial cells were mechanically removed from the vessels by gentle rubbing with a plastic rod in Hanks solution of the following compositions (composition in mmol/L): CaCl_2_ (1.6), MgSO_4_ (1.0), NaCl (145.0), KCl (5.0), NaH_2_PO_4_ (0.5), dextrose (10.0), and HEPES (10.0), maintained at pH 7.4 and constant temperature of 37 ± 1°C. The endothelial cells (1 × 10^6^ cells/mL) were divided in polypropylene tubes, centrifuged at 1500 rpm for 5 minutes, suspended in 500 *μ*L of Hanks solution containing 10 *μ*M DAF-2/DA (specific fluorescent dye for NO detection), and incubated in a humidified atmosphere of 5% CO_2_ in air at 37°C for 20 minutes. After incubation, the cells were treated with HEJ (EC_50_), Ach (EC_50_), or distilled water in the same volume and incubated again for 5 minutes. Then, [NO]_c_ levels were quantified by a flow cytometer (BD FACSCanto II, Biosciences, USA) recording 10,000 events for each five independent analyses.

### 2.7. Reagents

All chemicals of reagent grade (acetylcholine, ellagic acid, L-NAME, phenylephrine, and sodium nitroprusside) were obtained from Sigma (Sigma-Aldrich Inc., St. Louis, MO, USA). All other chemicals used in the present study were commercially available and of reagent grade. The purity of all substances was >97%. The concentrations given are the final concentrations in the bath solution.

### 2.8. Statistical Analysis

In the graphics, the data are presented as means ± SEM. The statistical analysis was performed using GraphPad Prism version 5.0 (GraphPad Software Corporation). Comparisons among groups were performed using ANOVA (post hoc test: Newman–Keuls), and values of *p* < 0.05 were considered to be significant.

## 3. Results and Discussion

### 3.1. Chemical Characterization of Extract

The concentration of total phenolic compounds in the HEJ (calculated on the total solid content in the extract) was 17.85%. In the HPLC analyses, HEJ showed the presence of ellagic acid (Rt: 7.826 min). The level of ellagic acid (chemical marker) found in HEJ was 0.23% (*w*/*w*), identified by comparison with external standard (Sigma-Aldrich Co., St. Louis, MO, USA). The ellagitannins present in foods change to free ellagic acid and its derivatives during digestion [18]. Ellagic acid has a high efficiency as an antioxidant compound due to the presence of several hydroxyl groups, which are responsible for the strong potential to donate a hydrogen atom and support the unpaired electron [19]. According to literature, the content of total ellagic acid in jabuticaba fruits (*M. cauliflora*) varied from 0.0215% to 0.311% (*w*/*w*). The level of ellagic acid found in HEJ was 0.23% (*w*/*w*); thus, the concentration of this compound in the HEJ is in agreement with the content in fruits [6]. There has been an increasing interest in this chemical marker due to its powerful antioxidant activities and other properties such as cardioprotective effects and angiotensin-converting enzymes [18, 20].

**Figure 1:**
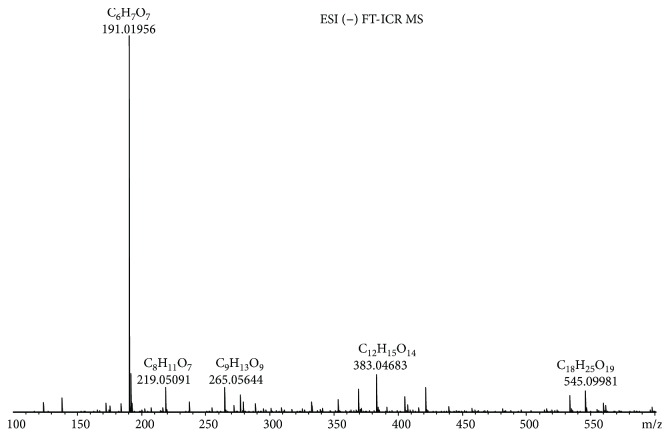
ESI(−) FT-ICR MS mass spectrum of the hydroalcoholic extract of jabuticaba (HEJ). The peaks show the phenolic compounds present in the jabuticaba extract. For peak identification, the molecular structure can be seen in Table 1.

We acquired the ESI(−) FT-ICR mass spectra of the jabuticaba berry extract (Figure 1). The ESI(−) FT-ICR mass spectra shows that the ions of *m*/*z* 191.01956, 219.05091, 265.05644, 383.04683, and 533.13619 are the most abundant species, detected as sodium adducts: [C_6_H_8_O_7_-H]^−^, [C_8_H_12_O_8_-H]^−^, [C_9_H_14_O_9_-H]^−^, [C_12_H_16_O_14_-H]^−^, and [C_18_H_30_O_18_-H]^−^, respectively. The majority compounds and derivatives in the HEJ were revealed by ESI(−) FT-ICR mass spectrometry as illustrated in Table 1.

In the one-dimensional spectrum, the NMR analyses identified five groups of signals representing phenolic compounds (200 *μ*g/mL), like anthocyanins, tannins, flavonoids, and phenolic acids. Coumarins were also observed in a very similar concentration (~200 *μ*g/mL). Other compounds were identified in the spectrum: rhamnose (390 *μ*g/mL), cinnamic acid (40 *μ*g/mL), fructose (170 *μ*g/mL), glycerol (2.17 mg/mL), lactate (120 *μ*g/mL), *α*,*β*-glucose (140 *μ*g/mL), and succinic acid (1.11 mg/mL).

We introduce the ESI(−) Fourier transform ion cyclotron resonance mass spectrometry (FT-ICR MS) combined with NMR to characterize HEJ without prior extraction or separation. FT-ICR MS routinely provides ultrahigh mass resolving power, *m*/Δm50% > 400,000, and mass accuracy better than 1 ppm. These high specifications mean that FT-ICR is ideal for analyzing complex mixtures [21]. Moreover, it becomes possible to assign molecular formulas (CcHhNnOoSs) unambiguously by mass measurement from singly charged ions such as [M + H]^+^, [M + Na]^+^, or [M − H]^−^, where “M” corresponds to neutral molecules. Furthermore, NMR methods provide information on a wide range of compounds present in the HEJ matrix in a single experiment, offering advantages in terms of simplicity of sample preparation and brevity of analyses while providing quantitative information about the compounds detected in the jabuticaba berry extract. This study demonstrates that HEJ contain high concentration of phenol compounds and possess high-quality antioxidant properties.

### 3.2. Antioxidant Activity

**Figure 2:**
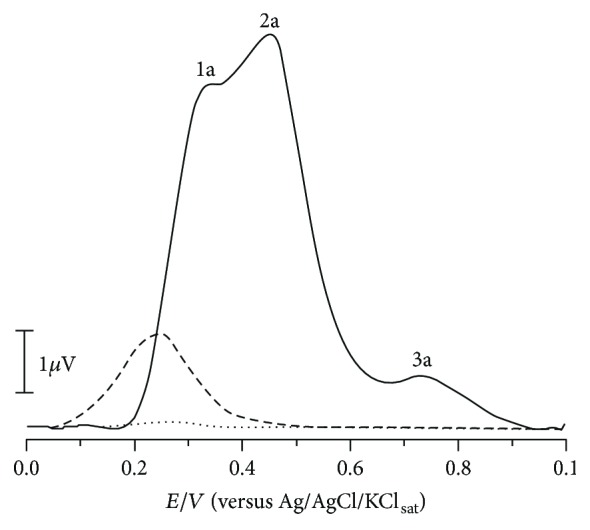
Average DP voltammograms obtained for 50 *μ*L of HEJ (12%; continuous line), standard ascorbic acid (positive control, 10 *μ*M; dashed line), and blank (dotted line) in 5 mL of 0.1 M phosphate buffer (pH 6.0) solution characterized at carbon paste electrodes (*Ø* = 2 mm). Other parameters included a pulse width of 5 mV, a pulse amplitude of 50 mV, and a scan rate of 5 mV·s^−1^.

From the DPV, it was possible to observe the presence of electroactive compounds in the HEJ, which presented three oxidation peaks, 1a, 2a, and 3a, at *E*_p1a_ ± 0.33 V, *E*_p2a_ ± 0.45 V, and *E*_p3a_ ± 0.71 V (Figure 2). It is well established that peaks below 0.5 V (pH 5.0) are related to compounds presenting high reducing power [17, 22].

Moreover, the high radical scavenging power of HEJ was also evidenced by the DPPH assay, in which it was found that each milligram of the HEJ present the antioxidant power equivalent to 7.08 mg gallic acid.

Antiradical compounds are normally reduced in living organisms at 0.8 V. Therefore, electroactive compounds exhibiting peak potentials lower than 0.7 V (pH 7.0) will be able to scavenge the free radicals. Furthermore, since ascorbic acid and tocopherol present reduction potential below 0.5 V (pH 7.0), the jabuticaba extract could restore such endogenous antioxidants [22, 23]. As antioxidants are electroactive compounds, the electroanalytical methods are indicated and very suitable to their study. It has been demonstrated that the HEJ present high antioxidant potential.

### 3.3. Cardiovascular Assessment and Body Weight

**Figure 3:**
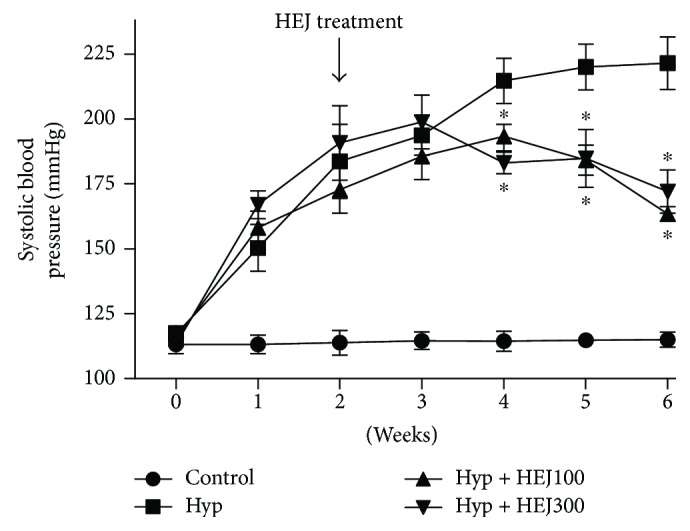
Systolic blood pressure (mmHg) measured by a tail-cuff method in the four experimental groups (control, hypertensive, hypertensive treated with HEJ (100 mg/kg/day), or hypertensive treated with HEJ (300 mg/kg/day)) during the study period (*n* = 5–7 per group). Data are shown as means ± SEM and were analyzed using one-way ANOVA followed by the post hoc Newman–Keuls test. ^∗^*p* < 0.05 versus hypertensives. From the first week, all groups are statistically different in relation to the control.

Before the experiments, the baseline SBP and body weight were similar in the four experimental groups. The L-NAME administration induced a rapid gradual rise in SBP, reaching 225.8 ± 11.2 mmHg in the sixth week (Figure 3, Table 2). In the L-NAME + HEJ- (100 and 300 mg/kg) treated group, SBP values were determined to decrease significantly at the fourth week of HEJ treatment in comparison with the those in the group receiving L-NAME alone. Alternatively, in the control group, SBP values remained stable all through the six-week period. No significant differences in body weight were observed among the experimental groups. Another condition closely related to hypertension is oxidative stress, which plays an important role in the pathogenesis of hypertension [24, 25]. The reactive oxygen species (ROS) react with NO, decreasing its bioavailability. It is probable that treatment with HEJ, which showed a high antioxidant capacity, could neutralize the ROS and increase the NO bioavailability, attenuating hypertension. Another possibility is the direct relaxing effect of the HEJ on vascular smooth muscle (as shown by our research group) [14], which could reduce the peripheral vascular resistance and, consequently, the blood pressure.

Treatment with HEJ also attenuated the increases in the heart rate (bpm) in hypertensive rats (Table 2). Tachycardia is a compensatory physiologic phenomenon that is a common phenomenon during hypertension [26].

In the heart and kidneys, the increase in blood pressure has a direct relationship to damage and hypertrophy, which is characterized by an increase in organ volume and weight [27]. The weight of the heart and kidneys also was measured after six weeks of experiments. Both organs in the rats treated with HEJ recovered the weight (hypertrophic index) raised by hypertension (Table 2). The antiproliferative [28] and anti-inflammatory [9] activity of jabuticaba fruit could be responsible for this cardiovascular and renal effects, since some lesions induced by hypertension are hypertrophic and inflammatory in origin.

### 3.4. Vascular Function

Short-term systemic blood pressure control is carried out by a sophisticated physiological system involving hormonal and neural regulations that strongly influence the blood vessel tone and heart function. Thus, a single substance that interferes with the function of arteries, veins, or the heart will quickly alter blood pressure [29].

**Figure 4:**
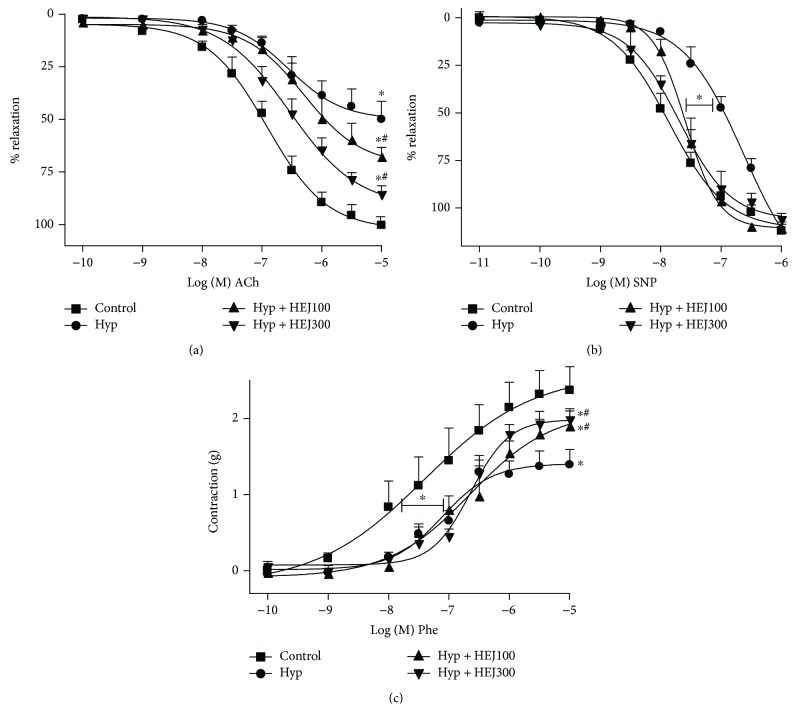
Vascular reactivity studies in aortic artery rings in the four experimental groups (control, hypertensive, hypertensive treated with HEJ (100 mg/kg/day) or hypertensive treated with HEJ (300 mg/kg/day)) after six-week treatment (*n* = 6 − 7 per group). (a) Endothelium-dependent relaxation in response to ACh. (b) Endothelium-independent relaxation in response to sodium nitroprusside (SNP). (c) Cumulative concentration-response curve to phenylephrine in arteries without the endothelium. Data are represented as mean ± SEM and were analyzed using one-way ANOVA followed by the post hoc Newman–Keuls test. ^∗^*p* < 0.05 versus control and ^#^*p* < 0.05 versus hypertensives.

The endothelium-dependent vasorelaxation and endothelium-independent vasorelaxation were induced by ACh and SNP, respectively, and can be observed in Figures 4(a) and 4(b). The hypertension was associated with the significant impairment of the ACh maximum response (50.1 ± 6.6%, *n* = 8) as compared to the control group (100.3 ± 4.1%, *n* = 8). The treatment with HEJ (100 and 300 mg/kg) significantly improved the relaxation by ACh to 67.7 ± 5.6% (*n* = 6) and 85.9 ± 5.3% (*n* = 6), respectively. The reduction of SBP by HEJ could be related to antioxidant-induced improvement of vascular relaxation. In isolated arteries, the endothelium-dependent relaxation stimulated by ACh was significantly impaired in the hypertensive group when compared to the control group, as reported in previous studies using hypertensive animals [30, 31]. Herein, the impaired endothelial reactivity in arteries of hypertensive animals was attenuated by the HEJ treatment in both doses (Figure 4(a)).

In the same way, the hypertension was also associated with the significant impairment in the pD_2_ (−log EC_50_) response to SNP (6.39 ± 0.39, *n* = 7), as compared to the control group (7.80 ± 0.14, *n* = 9). This fact also was observed by other works [32, 33]. The treatment with HEJ (100 and 300 mg/kg) significantly improved the relaxation (pD_2_ values) by SNP to 7.56 ± 0.12 and 7.60 ± 0.16, respectively. Some authors have attributed this impairment to an increased ROS production in the arteries from hypertensive rats, since NO reacts with ROS, reducing NO bioavailability [33, 34] (Figure 4(b)).

The contractile response to vascular tissues after adrenergic stimulation is generally controversial in arteries from hypertensive rats. There are doubts about whether the hypertension induces hypo- or hyperactivity to adrenergic contractile stimulus, since experiments have shown that vascular responsiveness in hypertensive rat arteries was increased [35], decreased [33], or unchanged [36]. In our work, the hypertension caused impairment in the contraction induced by Phe (adrenergic agonist) in arteries. The treatment with HEJ was able to restore and improve the constriction when compared to the hypertensive and control groups. The *E*_max_ observed for the hypertensive group was lower (1.39 ± 0.12 g, *n* = 7) than that observed for the control group (2.36 ± 0.31 g, *n* = 9). The treatment with HEJ (100 and 300 mg/kg) improved the contraction induced by Phe (E_max_) to 1.89 ± 0.17 g (*n* = 5) and 1.97 ± 0.19 g (*n* = 5), respectively (Figure 4(c)).

### 3.5. Cytosolic Concentration of NO ([NO]_c_) in Endothelial Cells

**Figure 5:**
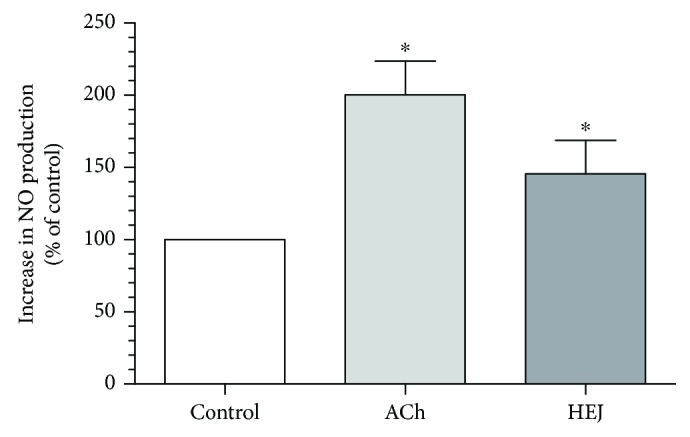
Effects of HEJ and ACh on [NO]_c_ production in endothelial cells obtained from the aorta of rats. After incubation with DAF-2/DA, the cells were treated with HEJ or ACh for 10 min and [NO]_c_ levels were quantified by a flow cytometer. Each bar presents mean ± SEM of each five independent analyses (^∗^*p* < 0.05 versus control. ANOVA followed by the post hoc Newman–Keuls test, *p* < 0.05).

Endothelial cells can modulate the vascular smooth muscle response to different contractile or relaxant stimuli [37]. Since it was first demonstrated that the acetylcholine-induced relaxation of contracted arteries is mediated by NO, the role of the endothelium in regulating the vascular tone has been well established. NO is a potent vasodilator, which lowers blood pressure by several cellular pathways [32, 33]. As shown in Figure 5, ACh promoted a significant increase of 203.3 ± 21.2% in the [NO]_c_ production in endothelial cells when compared to the basal levels of the control group. Similarly, the treatment with HEJ also promoted a significant increase in [NO]_c_ levels (148.9 ± 13.7%). Both stimuli increased NO production from the endothelial cells, highlighting the capacity of the HEJ to produce endothelial NO.

## 4. Conclusions

In conclusion, this study demonstrated that the extract obtained from the fruits of *M. cauliflora* presents a high antioxidant potential. The treatment with HEJ attenuated hypertension possibly improving the NO biodisponibility. The endothelium-dependent and endothelium-independent vascular contraction and relaxation were impaired by hypertension and improved after treatment with HEJ. Further, the HEJ has the ability to stimulate the production of NO from endothelial cells. Therefore, this work will contribute to the body of knowledge about jabuticaba-derived compounds and their use as a medicinal plant allied for cardiovascular and oxidative illness prevention and health.

## Figures and Tables

**Table 1:** Elemental compositions assigned to peaks in the negative-ion ESI FT-ICR mass spectrum of the hydroalcoholic extract of jabuticaba (HEJ).

Possible structures	Measured *m*/*z*	Molecular formula	Error (ppm)
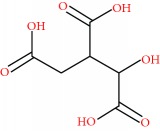	191.01956	[C_6_H_8_O_7_-H]^−^	0.87

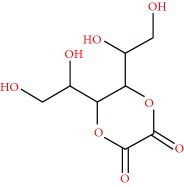	219.05091	[C_8_H_12_O_8_-H]^−^	0.55

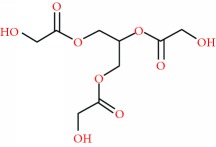	265.05644	[C_9_H_14_O_9_-H]^−^	0.26

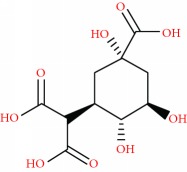	277.05492	[C_10_H_14_O_9_-H]^−^	0.86

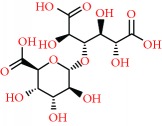	369.06751	[C_12_H_18_O_14_-H]^−^	0.14

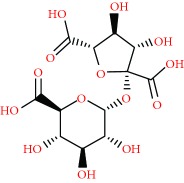	383.04683	[C_12_H_16_O_14_-H]^−^	0.28

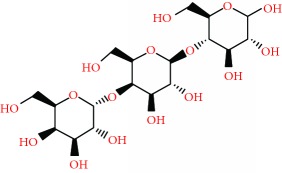	533.13619	[C_18_H_30_O_18_-H]^−^	0.48

**Table 2:** Systolic blood pressure and general parameters with the treatment with the hydroalcoholic extract of jabuticaba (HEJ).

Parameters	Control	Hypert	Hypert + HEJ100	Hypert + HEJ300
SBP (mmHg)	115.1 ± 5.8	225.8 ± 11.2^∗^	163.4 ± 8.7^∗^^#^	172.1 ± 9.3^∗^^#^
HR (beats/min)	364.2 ± 5.4	407.1 ± 13.5^∗^	370.5 ± 15.3^#^	368.7 ± 9.2^#^
Body weight (g)	308.8 ± 7.7	298.9 ± 6.3	302.5 ± 9.4	305.2 ± 9.9
Heart weight	0.291 ± 0.011	0.384 ± 0.025^∗^	0.339 ± 0.019^∗^^#^	0.349 ± 0.015^∗^^#^
Kidney weight	0.358 ± 0.011	0.405 ± 0.016^∗^	0.340 ± 0.021^#^	0.344 ± 0.008^#^

Systolic blood pressure (SBP), heart rate (HR) (beats/min), body weight (g), and heart and left kidney weight (% body weight) in control rats, hypertensive rats, and HEJ- (100 and 300 mg/kg) treated hypertensive rats. Data are represented as mean ± SEM (*n* = 5–7 rats) and were analyzed using one-way ANOVA followed by the post hoc Newman–Keuls test. ^∗^*p* < 0.05 versus control and ^#^*p* < 0.05 versus hypertensive.

## Abbreviations

ACh: Acetylcholine
HEJ: Hydroalcoholic extract of jabuticaba
L-NAME: N*ω*-Nitro-L-arginine methyl ester hydrochloride
NO: Nitric oxide
NOS: Nitric oxide synthase
Phe: Phenylephrine
ROS: Reactive oxygen specie
SBP: Systolic blood pressure
SNP: Sodium nitroprusside.
